# Characterization of 5-(2- ^18^F-fluoroethoxy)-L-tryptophan for PET imaging of the pancreas

**DOI:** 10.12688/f1000research.9129.2

**Published:** 2016-11-14

**Authors:** Ahmed Abbas, Christine Beamish, Rebecca McGirr, John Demarco, Neil Cockburn, Dawid Krokowski, Ting-Yim Lee, Michael Kovacs, Maria Hatzoglou, Savita Dhanvantari

**Affiliations:** 1 Department of Medical Biophysics, Western University, London, ON, N6A 5C1, Canada; 2 Metabolism and Diabetes Program, Lawson Health Research Institute, London, ON, N6A 4V2, Canada; 3 Imaging Program, Lawson Health Research Institute, London, ON, N6A 4V2, Canada; 4 Department of Genetics and Genome Sciences, Case Western Reserve University, Cleveland, OH, 44106, USA; 5 Department of Pathology and Laboratory Medicine, Western University, London, ON, N6A 5C1, Canada

**Keywords:** pancreas, beta cell, insulin, diabetes, PET, LAT1, Akita mice, ER stress, biomarker

## Abstract

*Purpose*: In diabetes, pancreatic beta cell mass declines significantly prior to onset of fasting hyperglycemia. This decline may be due to endoplasmic reticulum (ER) stress, and the system L amino acid transporter LAT1 may be a biomarker of this process. In this study, we used 5-(2- ^18^F-fluoroethoxy)-L-tryptophan ( ^18^F-L-FEHTP) to target LAT1 as a potential biomarker of beta cell function in diabetes.

*Procedures:* Uptake of ^18^F-L-FEHTP was determined in wild-type C57BL/6 mice by *ex vivo* biodistribution. Both dynamic and static positron emission tomography (PET) images were acquired in wild-type and Akita mice, a model of ER stress-induced diabetes, as well as in mice treated with streptozotocin (STZ). LAT1 expression in both groups of mice was evaluated by immunofluorescence microscopy.

*Results: *Uptake of ^18^F-L-FEHTP was highest in the pancreas, and static PET images showed highly specific pancreatic signal. Time-activity curves showed significantly reduced ^18^F-L-FEHTP uptake in Akita mice, and LAT1 expression was also reduced. However, mice treated with STZ, in which beta cell mass was reduced by 62%, showed no differences in ^18^F-L-FEHTP uptake in the pancreas, and there was no significant correlation of ^18^F-L-FEHTP uptake with beta cell mass.

*Conclusions:* ^18^F-L-FEHTP is highly specific for the pancreas with little background uptake in kidney or liver. We were able to detect changes in LAT1 in a mouse model of diabetes, but these changes did not correlate with beta cell function or mass. Therefore, ^18^F-L-FEHTP PET is not a suitable method for the noninvasive imaging of changes in beta cell function during the progression of diabetes.

## Introduction

In both Type 1 and Type 2 diabetes, the ability of the beta cells in the pancreatic islets of Langerhans to produce insulin is disrupted. There is an extensive preclinical period of time during which beta cell mass is significantly reduced prior to the onset of fasting hyperglycemia ^1^, and therefore, there have been several efforts to detect these changes non-invasively, with the hypothesis that disease onset may be delayed and/or halted. Such efforts have focused on engineering transgenic mice in which uptake of an imaging contrast agent is genetically enhanced in beta cells using the mouse insulin promoter ^2^ or on identifying biomarkers on the beta cell membrane that can be targeted by specific ligands that carry image contrast ^3, 4^.

We ^5^ and others ^6, 7^ have developed transgenic mouse models in which changes in beta cell mass can be imaged by positron emission tomography (PET) or bioluminescence. While using bioluminescence to image these same changes in beta cell mass is a more sensitive and cost-effective approach ^8^, PET has the advantage of being a clinical imaging modality, and our study showed that PET had the sensitivity to track changes in beta cell mass before the onset of fasting hyperglycemia in the context of a reporter gene. For a more clinically applicable approach, PET probes that target the vesicular monoamine transporter (VMAT2) ^3, 9^ and the glucagon-like peptide-1 receptor (GLP-1R) on the surface of the beta cell ^10, 11^ are available. While VMAT2 targeting had some initial success in humans, there appears to be heterogenous VMAT2 expression among beta cell population, thus limiting its use in detecting changes in beta cell mass ^12^. Targeting the GLP-1R using peptide analogs of GLP-1 and exendin-4 have been shown to be useful in imaging benign insulinomas and transplanted islets ^10, 13, 14^, but have very limited capacity to image beta cells in the native rodent pancreas ^15, 16^. This is due largely to unfavourable pharmacokinetics that result in accumulation of PET signal in the kidneys and liver ^17^, thus obscuring any signal that might have been emitted from pancreatic beta cells.

Although targeting these specific cell surface proteins for the molecular imaging of the beta cells has not been successful, it remains that such proteins would be useful targets for clinical PET imaging of beta cell mass or function. One possible biomarker that may reflect changes in beta cell function during the development of diabetes is the large neutral amino acid transporter, LAT1 (SLC7A5). LAT1 is a member of the solute carrier (SLC) transporter family of proteins that controls the uptake and efflux of solutes such as metabolites, ions, toxins and drugs. It is a Na ^+^-independent exchanger of amino acids with large neutral side chains, such as leucine, isoleucine, valine, tyrosine, tryptophan and methionine (reviewed in 18). LAT1 is expressed in the brain, testis and placenta, and is highly abundant in the blood-brain barrier ^19^, where it mediates the transport of metabolites and drugs into the CNS. Its expression is up-regulated in a variety of cancers, where it is thought to enhance the transport of amino acids required for nutritional support and signaling molecules for proliferation, and a number of PET imaging agents targeting LAT1 have been developed and tested for a range of human cancers ^20– 23^. One such tracer, [ ^18^F]-L-FEHTP, has been shown to specifically target LAT1 in cancer cells ^24^; remarkably, it also showed high uptake in the pancreas in mice, leading us to investigate its use in imaging beta cells.

LAT1 is expressed on the surface of pancreatic beta cells ^25^. Recently, alterations in islet LAT1 expression at the level of transcription have been linked to the development of beta cell dysfunction in a mouse model of diabetes in which a loss of regulation of amino acid transport leads to beta cell apoptosis ^26^. We therefore used the previously synthesized LAT1-targeted PET tracer, [ ^18^F]-L-FEHTP, and characterized its potential for the molecular imaging of beta cell function in diabetes.

## Materials and methods

### Synthesis and Labeling of ^18^F-L-FEHTP

^18^F-L-FEHTP was prepared by 2-step radiolabeling modeled after similar methods previously described ^24, 27^. ^18^F-fluoride was produced by ^18^O(p,n) ^18^F nuclear reaction bombarding enriched ^18^O-water in a cyclotron (PETtrace; GE). The fluoride was loaded onto an automated synthesis unit (Tracerlab FX _F-N_; GE) where it was trapped on an anion-exchange cartridge (QMA Light; Waters). The activity was eluted with a 1 mL solution of 1:4 water/ACN with Cryptand 222 (15 mg; ABX) and potassium carbonate (5 mg; Aldrich). The activity was dried under a stream of nitrogen with vacuum at 95°C. Acetonitrile (0.5 mL) was added and dried twice to remove residual water azeotropically. A 1 mL solution of ethylene di(p-toluenesulfonate) (10 mg, Aldrich) in acetonitrile was added and the mixture was heated for 6 min at 85°C. The reaction mixture was cooled and the ^18^F-labeled product was isolated by semi-preparative HPLC. The collected fraction was diluted in water and loaded onto a C-18 cartridge (Waters). The product was eluted in 2 mL of dimethyl sulfoxide (DMSO) and passed off of the automated synthesis unit into an adjoining hot cell for the second stage of the reaction.

5-Hydroxy-L-tryptophan disodium salt was prepared by combining 5-hydroxy-L-tryptophan (10 mg; Aldrich) with 2 equivalents of sodium methoxide in methanol (Aldrich) at room temperature. The methanol was removed by rotary evaporator (V-10; Biotage) and the salt was brought up in DMSO. The solution was transferred to a reaction vial under nitrogen. An aliquot of the ^18^F-labeled tosylate was added to the reaction vial and heated at 105 °C for 15 min. The reaction mixture was diluted with water and purified by semi-preparative HPLC, and the mobile phase from the collected fraction was removed by rotary evaporator. ^18^F-L-FEHTP product was dissolved in phosphate buffered saline with 8% ethanol and sterile filtered prior to use. Synthesis of the ^18^F-labelled tosylate on the automated synthesis unit yielded approximately 30% (decay corrected). The second reaction produced approximately 50% (decay corrected) isolated ^18^F-L-FEHTP in final buffered solution. The radiochemical purity of the final product was greater than 98%.

### PET imaging and image analysis

All mice were treated in accordance with the ethical guidelines set out by the Animal Use Subcommittee of the Canadian Council on Animal Care at Western University (protocol #2012-020). Both wild-type (wt) C57BL/6J male mice and C57BL/6J-Ins2C ^56^Y (Akita) male mice were obtained from Jackson Labs at 5 weeks of age, and imaging experiments were conducted at 6–7 weeks of age. For streptozotocin-treated mice, 8 female C57BL/6J mice were obtained from Jackson labs and randomly assigned to control (n = 4) or streptozotocin (STZ) treatment groups (single i.p. injection of 200 mg/kg, n = 4). Seven days post-injection, blood glucose readings were taken after fasting for 4 h using a One Touch Ultra glucometer (Lifescan Inc, Milpitas, CA). Diabetes was determined by glycemia ≥11 mmol/L. Imaging was conducted 8 days post-STZ injection.

All mice were fasted for 4h prior to the imaging session. Akita mice and corresponding controls were anesthetized with 0.5–2% isoflurane by inhalation, administered 6.45–15.8 MBq [ ^18^F]-L-FEHTP via tail vein injection and immediately placed on the scanner bed of a GE Healthcare Explore Vista DR PET scanner. STZ-treated mice and their corresponding controls were anesthetized, administered 9.5–11.1 MBq [ ^18^F]-L-FEHTP and immediately scanned in an Inveon preclinical PET scanner (Siemens Medical Solutions). PET image acquisition was conducted as described previously ^5^, with a dynamic scan for 1 h and a subsequent static scan for 30 min. Images were reconstructed and standardized uptake values (SUVs) were calculated as described previously ^5^, using regions of interest (ROIs) drawn through six image slices corresponding to the areas of the kidneys and pancreas.

### Biodistribution

To provide a pharmacokinetic profile of [ ^18^F]-L-FEHTP uptake, select organs, as well as urine and blood, were removed and weighed immediately after cessation of the imaging session, and counted in a high-purity Ge gamma counter, as described previously ^5, 11^. Data were calculated as % injected dose/g (%ID/g) tissue and all activity was decay-corrected to the time of injection.

### Immunofluorescence microscopy and image analysis

Immediately after counting, all pancreata from both wt and Akita mice were embedded in frozen tissue embedding gel (OCT, Fisher) for immunofluorescence microscopy analysis of LAT1. Serial sections were cut at 8 μm thickness, and three sections from each pancreas were selected for immunohistochemistry as described previously ^5^. Primary antibodies against insulin (polyclonal anti-guinea pig, 1:1000, Bachem, Cat # ab7842) and SLC7A5 (polyclonal anti-rabbit, 1:100, Sigma Chemical, Product# HPA052673) and respective Alexa secondary antibodies (Alexa 597, goat anti-guinea pig and Alexa 488, goat anti-rabbit) were used to visualize insulin and LAT1. Sections were also stained with Hoechst 33342 (Sigma) for nuclei visualization. Two to 10 fields of view per section were acquired at 20× magnification using Nikon NIS Elements v. BR 4.50.00 software and imported into ImageJ (Fiji) v.1.49v for analysis.

For image analysis, one ROI was drawn within each islet, two ROIs were drawn in the non-islet, non-ductal pancreatic areas and two ROIs were drawn in background regions of each field of view using the circle tool in ImageJ with an area of approximately 15000 pixels, as we have done previously ^28– 30^. Fluorescence intensities were calculated as corrected total cell fluorescence (CTCF) as follows:

*CTCF* = Raw Integrated density – (Area selected * mean background fluorescence)

### Beta-cell mass and morphometric analysis

Immediately following the static imaging session, STZ-treated mice were euthanized by CO _2_, and the dissected pancreata immediately fixed in 4% paraformaldehyde (PFA, Electron Microscopy Sciences, Hatfield, PA) for 24 h. Pancreata were prepared according to Beamish *et al*. ^31^. Seven micron thick cryosections were cut sequentially (Leica CM 1850 cryostat) from at least 3 layers, with an interval between each layer > 150 µm. Immunochemical staining followed Chamson-Reig *et al*. ^32^ using a human anti-mouse insulin primary antibody (1/200, Sigma Chemical, St Louis, MO), horse-anti-mouse secondary antibody (Vector Laboratories), and DAB chromagen (Biogenics Laboratories, Fremont, CA) according to manufacturer’s instructions. Three sections from different layers of pancreas were immunostained and analyzed. The entire pancreas section was imaged at 2.5X magnification, and insulin-positive cells were imaged at 40X magnification using Northern Eclipse software (v. 6.0, Empix Imaging, Mississauga ON Canada). Pancreas- and insulin-positive areas were measured by tracing using ImageJ v. 1.50b. Beta cell mass was calculated by dividing insulin-positive area by total pancreas area, then multiplying by pancreas weight.

### Statistical analyses

For biodistribution data, statistical significance between the wt and Akita organs was determined using one-way ANOVA followed by a Tukey post-hoc test. Differences in % ID/g between corresponding organs in the wt and Akita were compared with an unpaired one tailed Student’s t-test. For PET images, differences in SUVs were analysed using an unpaired one-tailed Student’s t-test. For microscopy images, differences in fluorescence values were determined using the Mann-Whitney U test. For correlation of SUV and beta cell mass, linear regression analysis was used. All statistical analyses were carried out on GraphPad Prism v. 6.01. Significance was set at p<0.05 for all experiments.

## Results

### *Ex vivo* biodistribution of ^18^F-L-FEHTP in Akita mice

**Figure 1.**
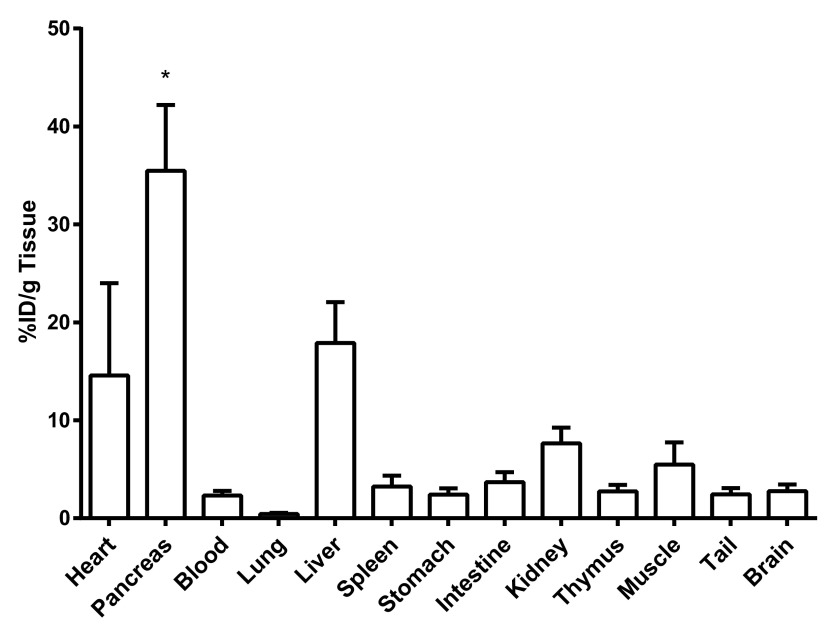
Uptake of ^18^F-L-FEHTP in wt mice. *Ex vivo* biodistribution was calculated for the indicated organs as % injected dose/g tissue (%ID/g tissue) at 1.5 h after injection. Values are given as mean ± SEM (n=6). * p< 0.05 compared to all other organs except heart and liver.

To determine ^18^F-L-FEHTP uptake by various tissues, *ex vivo* organ biodistribution was assessed in wt mice immediately after PET imaging ( Figure 1). Uptake in the pancreas was significantly greater (p<0.05) from all other tissues except heart (p = 0.485) and liver (p = 0.2029).

**Figure 2.**
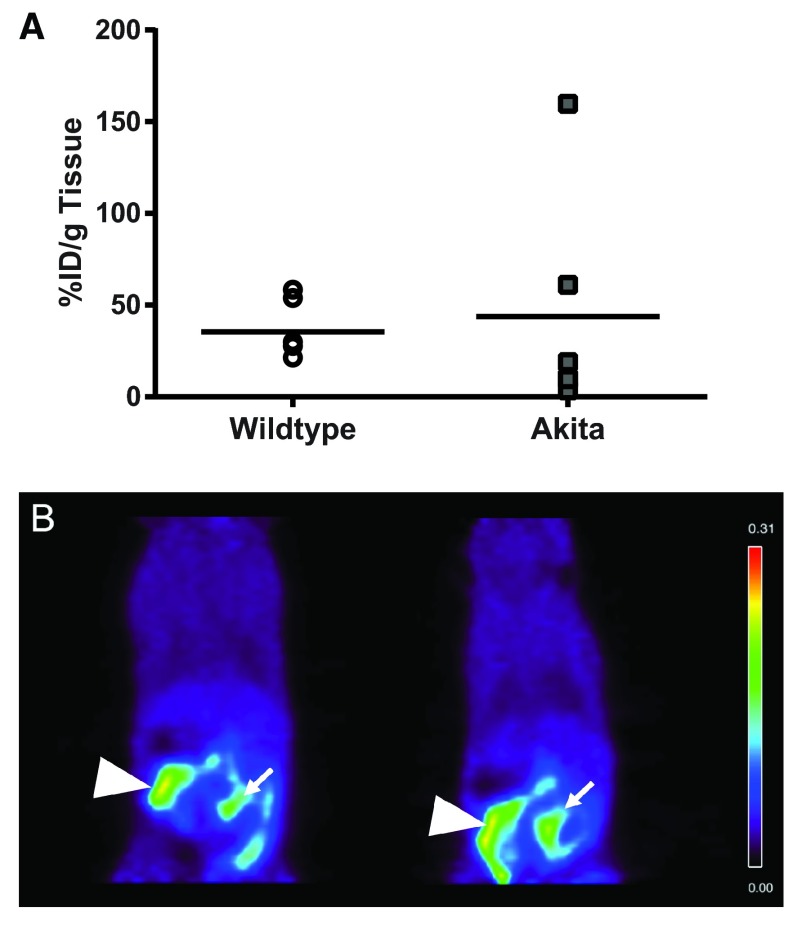
Pancreas-specific uptake of ^18^F-L-FEHTP in wt and Akita mice. **a**) *Ex vivo* tracer uptake was calculated for the pancreas of wt (n=6) and Akita (n=6) mice as % ID/g tissue at 1.5 h after injection. **b**) PET imaging of the pancreas after injection of ^18^F-L-FEHTP. One hour after injection, static images were acquired for 30 min. Representative images of wt (n=6) and Akita (n=5) mice show highly specific uptake in the pancreas with little detectable background uptake. Arrowheads indicate position of the pancreas. Arrows indicate position of spleen.

We then compared uptake of ^18^F-L-FEHTP in the pancreas of wt and Akita mice, which were previously reported to have higher LAT1 mRNA expression in pancreatic beta cells. There was notable variability in ^18^F-L-FEHTP uptake in the pancreata of Akita mice ( Figure 2A). The pancreas of the Akita mice group showed no significant difference in ^18^F-L-FEHTP uptake from the wt pancreas ( Figure 2A). *Ex vivo* biodistribution of all organs in Akita mice showed no significant differences in ^18^F-L-FEHTP uptake except the liver (p = 0.0144) ( Table 1).

**Table 1.** *Ex vivo* biodistribution of ^18^F-L-FEHTP in wt and Akita mice.

Organ	Akita	Wild-type
Heart	6.94±4.7	14.58±9.4
Pancreas	43.81±24.7	35.47±6.7
Blood	6.32±4.1	2.31±0.5
Lung	3.52±1.5	0.43±0.1
Liver	4.32±1.9	17.89±4.2	**
Spleen	16.57±13.4	3.25±1.1
Stomach	5.77±3.4	2.42±0.6
Intestine	3.76±1.4	3.69±1.0
Kidney	11.36±5.4	7.64±1.6
Thymus	12.06±9.9	2.74±0.7
Muscle	19.53±16.5	5.49±2.3
Tail	5.79±2.3	2.45±0.6
Brain	3.50±1.6	2.76±0.7

Values are expressed as %ID/g tissue, and are means ± SEM (n=6). ** p< 0.01 compared to wt.

### PET imaging: static scan analysis

In order to determine if ^18^F-L-FEHTP PET could detect changes in pancreatic LAT1 activity due to diabetes, static images were acquired for 30 min 1 h after ^18^F-L-FEHTP injections for both wt and Akita mice. The pancreas was clearly visualized with little detectable background uptake ( Figure 2B and Supplementary Figure 1). The SUVs (means ± SEM) calculated from pancreatic ROIs were 1.5 ± 0.04 and 1.3 ± 0.04 for wt and Akita mice, respectively, and these values were not significantly different.

### Immunofluorescence microscopy of LAT1 in pancreas tissue

**Figure 3.**
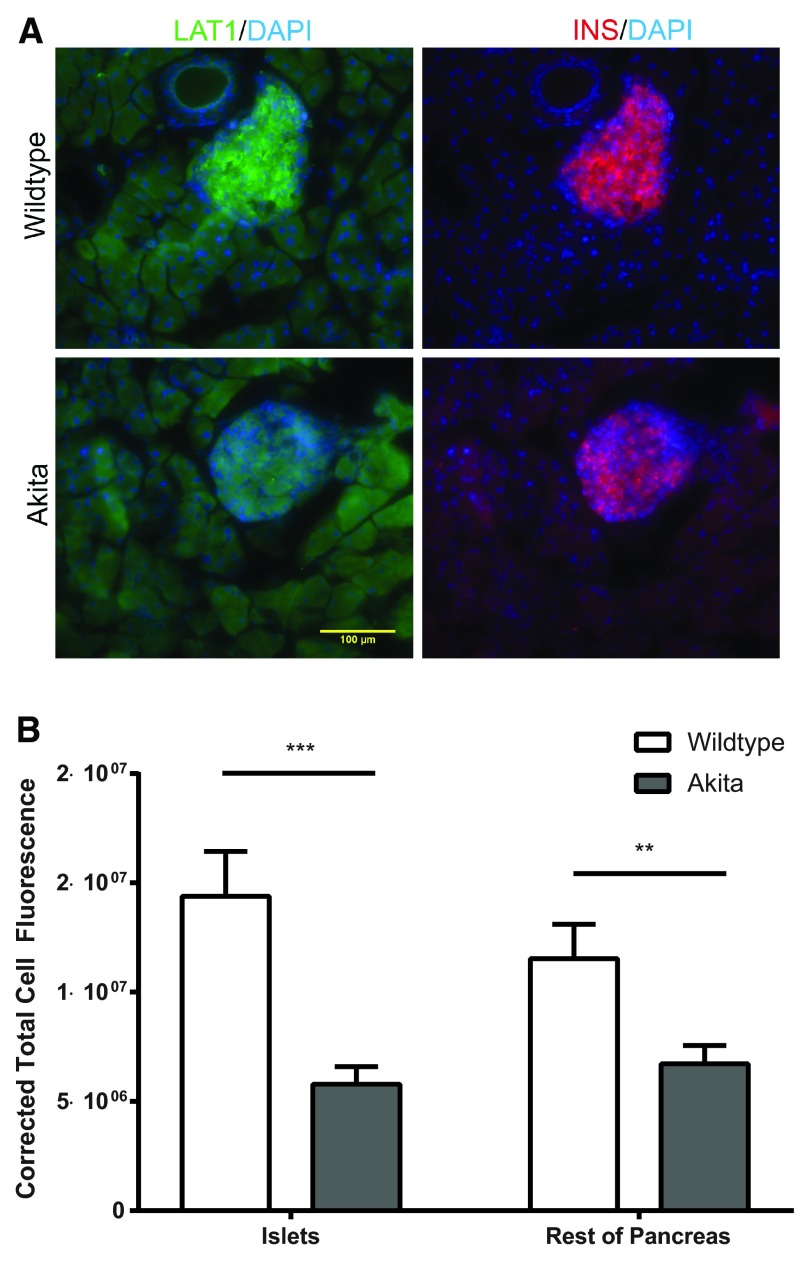
Immunofluorescence microscopy of LAT1 and insulin in the pancreas of wt and Akita mice. **a**) Representative fluorescence images of islets and surrounding tissue shows the distribution of LAT1 (green) and insulin (red). Nuclei are highlighted with DAPI staining (blue). **b**) Quantification of total corrected cell fluorescence of LAT1 immunoreactivity in islets and the rest of the pancreas. White bars represent values from wt mice, and grey bars represent values from Akita mice. Values are means ± SEM (n=6). * p<0.05 compared to corresponding wt values.

To investigate possible changes in LAT1 protein in pancreatic beta cells, immunofluorescence microscopy for LAT1 was conducted on pancreas sections from mice that had been imaged. Figure 3A shows representative immunofluorescence images for LAT1 and insulin in wt and Akita mice. Image analysis showed that there was significantly less LAT1 immunofluorescence in pancreatic islets from Akita mice, as well as in non-islet, non-ductal tissue ( Figure 3B). Additionally, there were no differences in total cell fluorescence between islets and the rest of the pancreas in both wt and Akita mice. This indicates that, at the protein level, LAT1 expression decreased in both islets and the rest of the pancreas in 6-week-old male Akita mice. Insulin immunofluorescence decreased by 62 ± 3% (p =0.0009) in Akita mice; however, total islet area did not differ between the two groups (12204 ± 1246 μm ^2^ vs. 11447 ± 671 μm ^2^, n = 6, p = 0.3).

### Dynamic scan analysis

**Figure 4.**
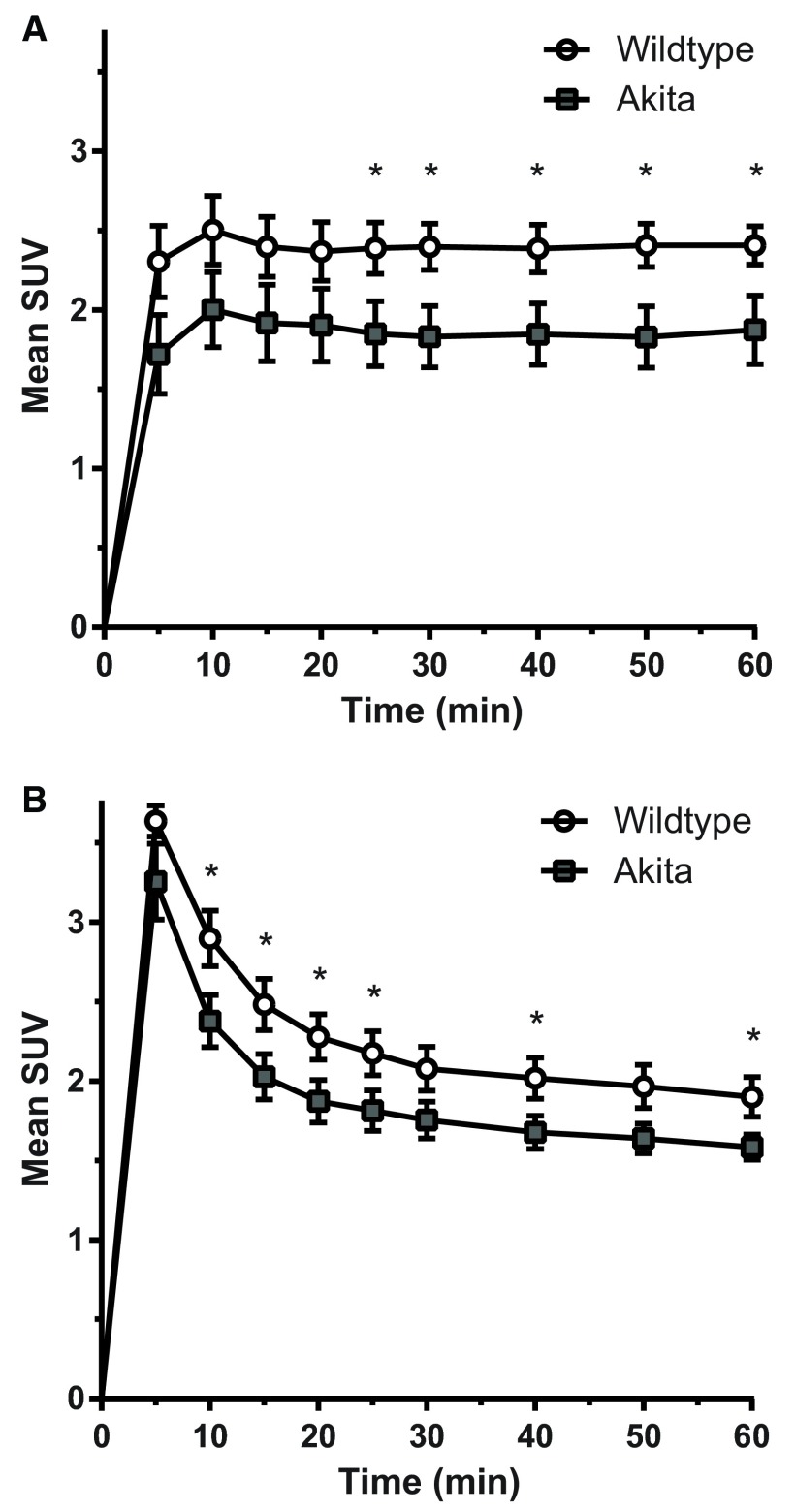
Time-activity curves (TACs) for ^18^F-L-FEHTP uptake in the pancreas of **a**) pancreas and **b**) kidney of wt and Akita mice. TACs are expressed as SUVs from regions corresponding to regions of interest from six image slices corresponding to the areas of the kidneys and pancreas. Values are means ± SEM (n=6 for wt, n=5 for Akita). * p<0.05 compared to wt.

Time-activity curves for ^18^F-L-FEHTP activity in the kidneys and pancreata of wt and Akita mice are shown in Figure 4. ROIs were assigned for the first and final frame (corresponding to 5 min and 60 min) around the kidneys and pancreas respectively. These frames were chosen because the tracer distributed to the kidneys initially ( Figure 4B) and then accumulated in the pancreas ( Figure 4A). Activity in the Akita group was consistently less than in the wt group for both organs ( Figure 4). In the pancreas, the Akita mice had significantly less ^18^F-L-FEHTP activity at 25 min (p = 0.0433), 30 min (p = 0.0245), 40 min (p = 0.0296), 50 min (p = 0.0192), and 60 min (p = 0.0348) ( Figure 4A).

### LAT1 imaging in STZ-treated mice

**Figure 5.**
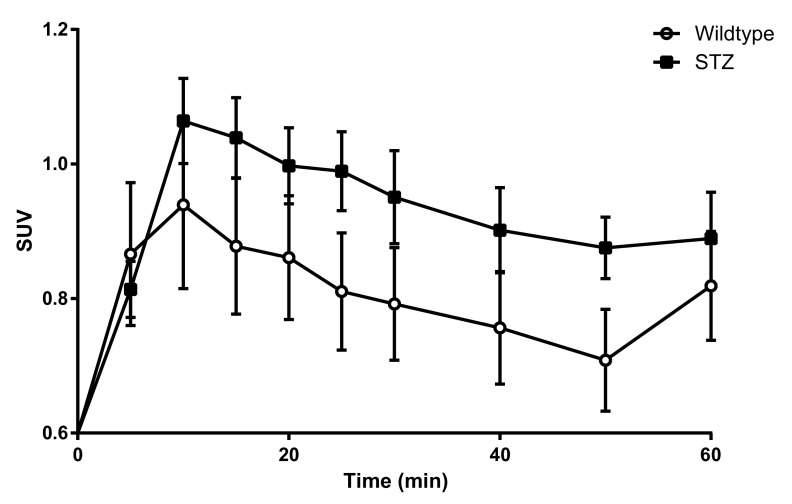
Time-activity curves for ^18^F-L-FEHTP uptake in the pancreas of wt and STZ-treated mice. TACs are expressed as SUVs from regions corresponding to regions of interest from six image slices corresponding to the pancreas. Values are means ± SEM (n=4). There were no significant differences at any of the time points.

**Figure 6.**
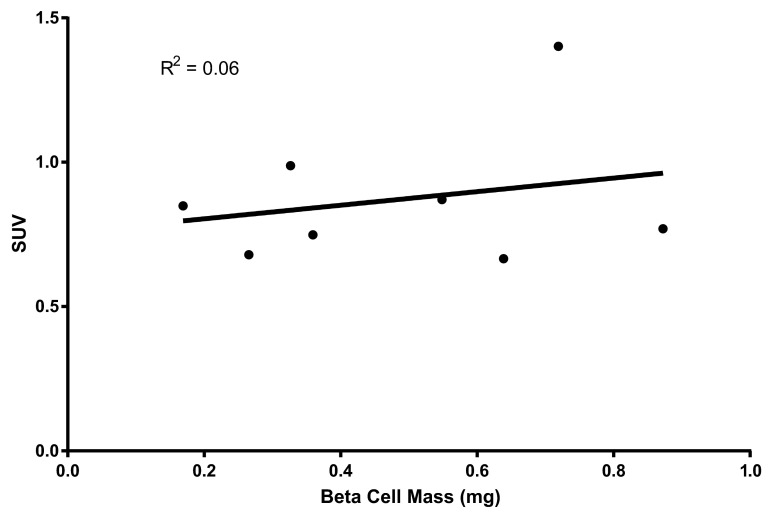
Correlation between beta cell mass and ^18^F-L-FEHTP uptake. SUVs from static scans from both wt and STZ-treated mice were plotted as a function of beta cell mass. Each point represents values from individual mice. Linear regression analysis showed no significant correlation between SUV and beta cell mass.

Imaging data in Akita mice seemed to suggest that the decrease in ^18^F-L-FEHTP correlated with a decrease in pancreatic LAT1 overall and was not specific to islets. We then examined ^18^F-L-FEHTP uptake in a known model of decreased beta cell mass to test the sensitivity of the tracer. Fasting blood glucose levels from STZ-treated mice were significantly higher than control (non-diabetic) animals (17.8 ± 3.7 mM vs 7.6 ± 0.2 mM, n = 4, p = 0.0161), which was commensurate with a 62% decrease in β-cell mass in STZ vs control mice (0.26 ± 0.04 mg vs 0.68 ± 0.08 mg, p < 0.0005). However, pancreatic uptake of ^18^F-L-FEHTP as assessed by both static (0.9 ± 1.7 vs 0.8 ± 0.1, p = 0.3) and dynamic PET scans ( Figure 5) was not different between the two groups, and there was no significant correlation (r = 0.2416) between pancreatic static SUVs and beta cell mass ( Figure 6).

Uptake of ^18^F-L-FEHTP in wt and Akita mice Raw biodistribution data for Figure 1 and Figure 2a. *Ex vivo* biodistribution values are given as % injected dose/g tissue (%ID/g tissue) for the organs indicated 1.5 h after injection. Click here for additional data file. Copyright: © 2016 Abbas A et al. 2016 Data associated with the article are available under the terms of the Creative Commons Zero "No rights reserved" data waiver (CC0 1.0 Public domain dedication).

Immunofluorescence microscopy of LAT1 Raw CTCF data for Figure 3b. Total corrected cell fluorescence of LAT1 immunoreactivity in islets (CTIF) and the rest of the pancreas (CTAF) in wt and Akita mice. Click here for additional data file. Copyright: © 2016 Abbas A et al. 2016 Data associated with the article are available under the terms of the Creative Commons Zero "No rights reserved" data waiver (CC0 1.0 Public domain dedication).

Time-activity values for ^18^F-L-FEHTP uptake in the pancreas and kidney of wt and Akita mice Raw SUV data for Figure 4 and Figure 6. Values are expressed as SUVs from regions corresponding to regions of interest from six image slices corresponding to the areas of the kidneys and pancreas. Click here for additional data file. Copyright: © 2016 Abbas A et al. 2016 Data associated with the article are available under the terms of the Creative Commons Zero "No rights reserved" data waiver (CC0 1.0 Public domain dedication).

Time-activity values for ^18^F-L-FEHTP uptake in the pancreas of wt and STZ-treated mice Raw SUV data for Figure 5. Values are expressed as SUVs from regions corresponding to regions of interest from six image slices corresponding to the pancreas. Click here for additional data file. Copyright: © 2016 Abbas A et al. 2016 Data associated with the article are available under the terms of the Creative Commons Zero "No rights reserved" data waiver (CC0 1.0 Public domain dedication).

Beta cell mass and ^18^F-L-FEHTP uptake Raw BCM data for Figure 6. Uptake is given as SUVs from static scans from both control and STZ-treated mice. Click here for additional data file. Copyright: © 2016 Abbas A et al. 2016 Data associated with the article are available under the terms of the Creative Commons Zero "No rights reserved" data waiver (CC0 1.0 Public domain dedication).

## Discussion

The PET tracer, ^18^F-L-FEHTP, is known to specifically target the amino acid transporter LAT1. Since the expression of the mRNA for LAT1 shows dramatic changes in pancreatic islets during the development of diabetes in the Akita mouse model ^26^, we used ^18^F-L-FEHTP in an effort to track these changes *in vivo*, and hypothesized that ^18^F-L-FEHTP uptake could be a surrogate measure for beta cell function. We also tested the hypothesis that ^18^F-L-FEHTP uptake could be an *in vivo* measure of beta cell mass by examining uptake in STZ-treated mice, a model of beta cell depletion. Interestingly, PET images of ^18^F-L-FEHTP uptake showed a strong signal in the pancreas, with minimal background uptake in the kidneys or liver. In male Akita mice, time-activity curves showed significantly reduced uptake of ^18^F-L-FEHTP, and immunofluorescence microscopy revealed a significant reduction in LAT1 in islets and the rest of the pancreas. However, there was no correlation of ^18^F-L-FEHTP uptake with beta cell function, nor with beta cell mass in STZ-treated mice. We showed that LAT1 was expressed throughout the pancreas, which may lead to challenges in detecting changes in LAT1 only in the islets, as they comprise 1% of total pancreatic volume.

Several ^18^F-labelled amino acid analogues have been developed for the targeting of LAT1 in cancer tumors. These tracers are based on analogues of L- and D-tyrosine ^20, 21^, or a racemic mixture of fluoropropyl tryptophan ^22^, as well as the fluoro-ethoxy analogue used in the present study, ^18^F-L-FEHTP ^24^. We chose ^18^F-L-FEHTP as a PET tracer that targets LAT1 since the biodistribution data in mice indicated the highest uptake in non-tumor tissue was in the pancreas, and the PET images in this study did not show appreciable background signal. Indeed, our own images show accumulation of the PET signal specifically in the area of the pancreas, with little background uptake in the kidneys or liver (although, uptake in the intestine and spleen cannot be ruled out). As well, uptake, efflux and metabolism of ^18^F-L-FEHTP have been very well characterized. 5-hydroxy-L-tryptophan is decarboxylated by amino acid decarboxylase, but ^18^F-L-FEHTP was shown to be very stable in cells with high amino acid decarboxylase activity, with no evidence of metabolite production *in vivo* ^24^. Therefore, we chose ^18^F-L-FEHTP due to its extensive characterization *in vitro* and *in vivo*.

The C96Y mutation in the mouse *Ins2* gene that characterizes the Akita mouse has been shown to induce beta cell apoptosis by failing to exit the ER and inducing ER stress ^33^, and this same mutation has been implicated in the development of permanent neonatal diabetes in humans ^34^. As proposed by Krokowski *et al.* ^26^, prolonged ER stress caused by induction of protein synthesis in beta cells involves increased amino acid flux through a network of transporters that leads to increased tRNA charging with LAT1 substrates such as leucine and tryptophan. In particular, LAT1 mRNA levels were significantly up-regulated during translational recovery in islets from 6-week-old male Akita mice, leading to the hypothesis that changes in LAT1 in islets could serve as a diagnostic biomarker for the early development of diabetes. Therefore, we reasoned that ^18^F-L-FEHTP would be a surrogate marker for the decline in beta cell function resulting from prolonged ER stress. In the present study, we first conducted PET imaging studies using the Akita mouse model. However, our PET imaging results suggest that ^18^F-L-FEHTP uptake was decreased in Akita mice, and immunofluorescence microscopy suggests that islet LAT1 also decreased.

It is possible that the decrease ^18^F-L-FEHTP uptake may be a function of a decline in beta cell mass. However, our results show no decrease in islet area, although there is a significant decrease in insulin immunofluorescence within islets. There is some conflicting evidence on changes in beta cell mass in Akita mice: in 8-week-old male mice, there is roughly a 50% decrease ^35^, while in 8-week-old female mice, there is no change, despite the sharp reduction in islet insulin content ^36^. Our results indicate that, in 6-week-old Akita male mice in our facility, ongoing ER stress is diminishing insulin synthesis, but has not yet affected islet size. Additionally, our analysis shows that there is no correlation between blood glucose levels and ^18^F-L-FEHTP uptake, indicating that imaging changes in LAT1 is not a surrogate measure for beta cell function.

Since islet area did not change in our Akita mice, we tested ^18^F-L-FEHTP uptake in a model of diabetes in which a dramatic decrease in beta cell mass is well documented. When treated with a single injection of 200 mg/kg STZ, female C57BL/6 mice showed a > 60% decrease in beta cell mass. However, there was no corresponding decrease in ^18^F-L-FEHTP uptake as assessed by either dynamic or static SUV analysis. Therefore, despite the loss of beta cells and associated LAT1 expression, no change in ^18^F-L-FEHTP uptake could be detected, leading us to conclude that ^18^F-L-FEHTP uptake is not a surrogate measure of changes in beta cell mass during the progression of diabetes.

Is ^18^F-L-FEHTP uptake a function of LAT1 expression? Our results show that both ^18^F-L-FEHTP uptake and LAT1 immunofluorescence are decreased in pancreatic tissue from Akita mice, so it appears that imaging with ^18^F-L-FEHTP can report LAT1 activity. However, LAT1 mRNA levels are increased in Akita islets ^26^, indicating that there may be an additional level of control in the expression of LAT1 in ER stress-dependent diabetes. There is evidence that LAT1 and other transporters are regulated differentially at the transcriptional and post-translational levels. Glucose deprivation increases LAT1 mRNA and protein expression and [ ^3^H]Leu transport activity in retinal endothelial cells ^37^. Interestingly, high glucose levels did not change LAT1 mRNA levels, indicating that hyperglycemia may regulate LAT1 at a post-translational level. Another study suggests a mechanism for post-translational regulation, as LAT1 localization to the plasma membrane is impaired by hyperoxia in alveolar epithelial cells ^38^, indicating that stress conditions can induce improper LAT1 trafficking, resulting in loss of transporter activity. Finally, another amino acid transporter, system A/SNAT2 (SLC38A2), is regulated differentially at the transcriptional and post-translational levels. Amino acid starvation and hypertonicity increase SNAT2 mRNA; however, stress conditions promote the proteasome-dependent degradation of SNAT2 ^39, 40^. It is possible that LAT1 in beta cells could be differentially regulated at the transcriptional and post-translational level in a similar manner, with nutrient availability and/or signaling determining the trafficking or degradation of LAT1 protein.

Since LAT1 is expressed in both the endocrine and exocrine compartments of the pancreas, the signal is likely due to uptake of ^18^F-L-FEHTP in both compartments. It was tempting for us to test the sensitivity of ^18^F-L-FEHTP in a model of pancreatic cancer. However, although the expression of many amino acid transporters do change in caerulein-induced pancreatitis, *slc7A5*/LAT1 expression/immunoreactivity does not ^41, 42^. So although ^18^F-L-FEHTP is taken up and retained in certain types of cancers ^24^, it is not an appropriate imaging probe for the detection of pancreatic cancer.

In order to differentiate islet uptake from acinar uptake, it has been estimated that the signal of any imaging probe must be 1000-fold higher in beta cells than in the surrounding acinar/ductal cells ^43^, due to the fact that beta cells comprise <1% of pancreatic volume. Our study clearly demonstrates that this is not the case for LAT1. The data for other presumptive beta cell imaging targets, such as VMAT2, GLP-1R, and the 5-HT metabolic pathway in human islets, are limited. A recent study showed that mRNA levels of VMAT2 in human pancreas were 500-fold higher in islets compared with exocrine cells ^44^; however, there was still significant uptake of [ ^18^F]FP-(+)-DTBZ in patients with T1D and no residual beta cell function. Using autoradiography, it has been reported that there is about 2X as many GLP-1 receptors in human islets than in acinar cells ^45^. We have shown the presence of GLP-1R in glucagon- and amylase-positive cells ^11^ and there is evidence for low-level expression of GLP-1R in exocrine pancreas. Finally, uptake of [ ^11^C]-hydroxytryptophan, postulated to be a surrogate measure of beta cell mass by targeting the serotonergic pathway, is 14 times higher in human islets than in exocrine cells ^46^, and there is a small but significant decrease in uptake in patients with T1D. Therefore, there is still some promise of a non-invasive method to image changes in beta cell function and mass during the progression of diabetes. Further characterization of ^18^F-L-FEHTP uptake and retention in human islets, and not in rodent-derived cell lines or islets, may be required.

## Conclusions

In the present study, we have shown specific accumulation of ^18^F-L-FEHTP in the pancreata of mice, with minimal background signal from the kidneys, liver or intestine. Such clear visualization of the pancreas using a targeted PET agent in mice has not previously been achieved. The PET signal represents the total integrated tracer uptake in the pancreas, and we could not detect changes in beta cell function or mass using our mouse models of Type 1 diabetes.

## Data availability

The data referenced by this article are under copyright with the following copyright statement: Copyright: © 2016 Abbas A et al.

Data associated with the article are available under the terms of the Creative Commons Zero "No rights reserved" data waiver (CC0 1.0 Public domain dedication). 

F1000Research: Dataset 1. Uptake of ^18^F-L-FEHTP in wt and Akita mice, 10.5256/f1000research.9129.d130932 ^47^

F1000Research: Dataset 2. Immunofluorescence microscopy of LAT1, 10.5256/f1000research.9129.d129356 ^48^

F1000Research: Dataset 3. Time-activity values for ^18^F-L-FEHTP uptake in the pancreas and kidney of wt and Akita mice, 10.5256/f1000research.9129.d129357 ^49^

F1000Research: Dataset 4. Time-activity values for ^18^F-L-FEHTP uptake in the pancreas of wt and STZ-treated mice, 10.5256/f1000research.9129.d129358 ^50^

F1000Research: Dataset 5. Beta cell mass and ^18^F-L-FEHTP uptake, 10.5256/f1000research.9129.d129359 ^51^

## Supplementary material

**Supplementary Video 1.**

3-D renderings of the representative images from **(a**) wt (n=6) and **(b**) Akita (n=5) mice which show highly specific uptake in the pancreas with little background uptake.
