# Supplementary material for: Characterization of 5-(2- 18F-fluoroethoxy)-L-tryptophan for PET imaging of the pancreas
Source: F1000Res. 2016 Nov 14;5:1851. Originally published 2016 Jul 27. [Version 2] doi: 10.12688/f1000research.9129.2 (PMC5112576; doi:10.12688/f1000research.9129.2)
Supplement: Peer review history

# Peer review: Referee response for version 2

**DOI:** 10.5256/f1000research.10835.r17569

**Published:** 2016-11-15

**Authors:** John Virostko

**Affiliations:** 1 Department of Radiology and Radiological Sciences, Vanderbilt University Medical Center, Nashville, TN, USA

All reviewer concerns have been addressed in the revised manuscript. This reviewer is pleased to see negative results being published in order to move the field forward.

I have read this submission. I believe that I have an appropriate level of expertise to confirm that it is of an acceptable scientific standard.

# Peer review: Referee response for version 1

**DOI:** 10.5256/f1000research.9823.r15268

**Published:** 2016-08-16

**Authors:** Christopher Phenix

**Affiliations:** 1 Department of Chemistry, University of Saskatchewan, Saskatoon, SK, Canada

The authors are developing a PET method using the tracer ^18^F-L-FEHTP, to detect changes in beta cell mass and/or function through imaging the expression of the LAT1 receptor within pancreas to identify those individuals at high risk of developing diabetes.  Although they find that the tracer is capable of revealing lower LAT1 expression in Akita mice (but not STZ treated mice), high uptake in other types of pancreatic cells may circumvent the use of ^18^F-L-FEHTP to image beta cell mass and function.

1. In the introduction, the authors mention another study that they published where PET was used to successfully image changes in beta cell mass prior to fasting hyperglycemia. I think it's important, for the context and rationale of this work, to explain why a different tracer was needed for this work since as written it appears they already have a good tracer (although reading the reference would make it clear that they actually imaged a reporter gene).
2. In the introduction, the sentence that discusses VMAT2 expression in other tissues is incomplete. Many PET tracers that image receptor expression are expressed in a variety of tissues and this does prevent their use. A bit more information should be added at the end of the sentence to help the reader understand why VMAT2 expression in other tissues resulted in such poor PET images.
3. The second image in Figure 2B has the arrow pointing into a location without any tracer uptake. I don’t think that is intentional and it is close to an area with high uptake. Although slight, the arrow should be moved a bit to clearly point out the pancreas in that specific image.  In addition, the figure would be generally improved by labelling all organs that demonstrate high tracer uptake in the PET images.
4. Would it have been reasonable to perform statistical analysis to determine if there is a potential outlier in the Akita mouse data (with very high pancreatic uptake) as shown in Figure 2A?
5. In paragraph 1 of the discussion, use of the word “amazingly” seems inappropriate in the paper.  It is established that LAT1 is highly expressed in the pancreas and high uptake was previously reported for this tracer by Kramer *et al*, (2012) so I wasn’t really “amazed” by the high uptake. The uptake in the pancreas is indeed impressive however.
6. In agreement with Dr. Virostko, I am a bit confused why there are dramatic changes in the % injected dose/g values measured in pancreas vs the kidney from the biodistribution data but much smaller relative changes in the SUV values obtained from the PET data. Can the authors explain this as I could be missing something? At least a comment in the text would be valuable.
7. Is there really no significant differences in uptake between the pancreas and blood shown in Figure 1?

I have read this submission. I believe that I have an appropriate level of expertise to confirm that it is of an acceptable scientific standard.

## References

[1] KrämerSDMuLMüllerAKellerCKuznetsovaOFSchweinsbergCFranckDMüllerCRossTLSchibliRAmetameySM: 5-(2-18F-fluoroethoxy)-L-tryptophan as a substrate of system L transport for tumor imaging by PET. *J Nucl Med*.2012;53(3) : 10.2967/jnumed.111.096289 434-42 10.2967/jnumed.111.096289 22331220

# Response

**Published:** 2016-10-31

**Authors:** Ahmed Abbas

**Affiliations:** University of Western Ontario, Canada

1. We apologize if the rationale of the study was not clear. We have added “in the context of a reporter gene” to line 14 of the Introduction to clarify the need to produce a clinically applicable PET tracer.
2. We have clarified our presentation of the VMAT2 findings.
3. We have adjusted the arrowhead to indicate a region of high uptake and indicated the location of the spleen.
4. We have tested for the presence of outliers in the Akita pancreas bioD (Figure 2A) as suggested. Based on Grubb’s test for outliers (alpha = 0.05), there are no outliers. Also, all values are within 2 standard deviations of the mean.
5. We have replaced “amazingly” with “interestingly”
6. See response to Dr. Virotsko’s comment.
7. We have run the statistical test again and confirmed that there is no significant difference in tracer uptake between pancreas and blood.

# Peer review: Referee response for version 1

**DOI:** 10.5256/f1000research.9823.r15266

**Published:** 2016-07-29

**Authors:** John Virostko

**Affiliations:** 1 Department of Radiology and Radiological Sciences, Vanderbilt University Medical Center, Nashville, TN, USA

This manuscript describes the use of the PET tracer [18F]-L-FEHTP in an attempt to image beta cell mass. Pancreatic uptake of the tracer is high. However, while experiments in Akita mice show a slight (but highly variable) decline in pancreatic [18F]-L-FEHTP uptake, there is no change in STZ-treated animals. The authors demonstrate that the target receptor, LAT1, is expressed throughout the pancreas, and is not confined to islets.

Comments

1. As biodistribution studies were performed immediately following the cessation of PET imaging, why does the biodistribution data (Figure 2A) not match the end of the TAC curve (Figure 4A)? This biodistribution data does not support the conclusion of ‘significantly reduced 18F-L-FEHTP uptake in Akita mice.’
2. In Figure 2 is all activity coming from the pancreas? It appears that there may be PET signal from other organs – is the spleen visible on these images?
3. Figure 4 caption states: ‘Time-activity curves (TACs) for 18F-L-FEHTP uptake in the pancreas of **a**) wt and **b**) Akita mice.’ However, I believe the figures show the pancreas TAC in panel A and the kidney TAC in panel B. The text under the heading ‘Dynamic scan analysis’ needs to be edited to correctly reference these figures.
4. It is interesting that the kidney of Akita mice also exhibits a statistically significant decline in tracer uptake. Is there known LAT expression in the kidney that would explain this finding?
5. The authors state that: ‘Such clear visualization of the pancreas using a targeted PET agent has not previously been achieved.’ Does the level of pancreatic uptake exceed that previously achieved with C11-acetate, another tracer with high pancreas uptake?
6. Figure 5: Connecting the points in the TAC would improve consistency with previously presented TACs.

I have read this submission. I believe that I have an appropriate level of expertise to confirm that it is of an acceptable scientific standard, however I have significant reservations, as outlined above.

# Response

**Published:** 2016-10-31

**Authors:** Ahmed Abbas

**Affiliations:** University of Western Ontario, Canada

1. This is an excellent question and one which everyone who characterizes a new PET tracer should think about. First, one difference between calculation of biodistribution and SUV data is that SUV is normalized to body weight, while biodistribution is normalized to organ weight. Second, SUVs calculated in the context of a dynamic scan gives an idea of tracer flux through the tissue, while bioD is a measure of the activity in each organ in the absence of blood circulation. These differences may account for the mismatch in the final time point of the TAC and the biodistribution results. These differences may also explain why liver uptake appears high in the bioD, but the liver signal cannot be discerned in the PET images.
2. The supplementary video shows that there was uptake in other organs (which do not appear in the images shown in figure 2). We agree that accumulation of activity in the spleen cannot be ruled out, and have inserted this comment in the Discussion (paragraph 2).
3. We thank Dr. Virotsko for alerting us to this error, and we have corrected the section under Dynamic Scan Analysis.Literature evidence (Fotiadis, *et al.* Reference #18) does not support LAT1 renal expression.
4. We have inserted the words “in mice” to reflect the fact that we are comparing our results to probes that target specific biomarkers (GLP-1R, VMAT2) in mouse pancreas. The key word in our statement is “targeted”. 11C-acetate does not target a specific mechanism, as its means of uptake and retention are not known. Studies of 11C-acetate have been done exclusively on humans and it is difficult to compare SUVs of 18F-FEHTP in mice and 11C-acetate in humans. The mean SUV of 11C-acetate activity in normal human pancreas is reported at 11.9±4.3 (Song *et al.* 2009, Nuc. Med. Commun. 30:462-465, DOI 10.1097/MNM.0b013e32832aa7ce), and our mean reported activity of 18F-FEHTP in WT C57Bl/6 male mice is 1.5±0.04.
5. We thank Dr. Virotsko for this suggestion and have connected the points in the TAC in Figure 5.
